# Estimates of Autozygosity Through Runs of Homozygosity in Farmed Coho Salmon

**DOI:** 10.3390/genes11050490

**Published:** 2020-04-30

**Authors:** Grazyella M. Yoshida, Pablo Cáceres, Rodrigo Marín-Nahuelpi, Ben F. Koop, José M. Yáñez

**Affiliations:** 1 Facultad de Ciencias Veterinarias y Pecuarias, Universidad de Chile, Santiago 8820808, Chile; grazyoshida@hotmail.com (G.M.Y.); pblo.caceres@gmail.com (P.C.); ro.nahuelpi@hotmail.es (R.M.-N.); 2 Department of Biology, Centre for Biomedical Research, University of Victoria, Victoria, BC V8W 2Y2, Canada; bkoop@uvic.ca; 3 Nucleo Milenio INVASAL, Concepción 4030000, Chile

**Keywords:** admixture, autozygosity, inbreeding, run of homozygosity, *Oncorhynchus kisutch*, runs of homozygosity, pedigree

## Abstract

The characterization of runs of homozygosity (ROH), using high-density single nucleotide polymorphisms (SNPs) allows inferences to be made about the past demographic history of animal populations and the genomic ROH has become a common approach to characterize the inbreeding. We aimed to analyze and characterize ROH patterns and compare different genomic and pedigree-based methods to estimate the inbreeding coefficient in two pure lines (POP A and B) and one recently admixed line (POP C) of coho salmon (*Oncorhynchus kisutch*) breeding nuclei, genotyped using a 200 K Affymetrix Axiom^®^ myDesign Custom SNP Array. A large number and greater mean length of ROH were found for the two “pure” lines and the recently admixed line (POP C) showed the lowest number and smaller mean length of ROH. The ROH analysis for different length classes suggests that all three coho salmon lines the genome is largely composed of a high number of short segments (<4 Mb), and for POP C no segment >16 Mb was found. A high variable number of ROH, mean length and inbreeding values across chromosomes; positively the consequence of artificial selection. Pedigree-based inbreeding values tended to underestimate genomic-based inbreeding levels, which in turn varied depending on the method used for estimation. The high positive correlations between different genomic-based inbreeding coefficients suggest that they are consistent and may be more accurate than pedigree-based methods, given that they capture information from past and more recent demographic events, even when there are no pedigree records available.

## 1. Introduction

Coho salmon (*Oncorhynchus kisutch*) is one of the six Pacific salmon species which can be found in North America and Asia [1]. In Chile, coho salmon farming was established during the late 1970s, date in which about a half of million eggs were imported from Oregon (USA) and the Kitimat river (British Columbia, Canada), giving origin to the basis of the commercial stocks in the country [2,3]. The first coho salmon breeding program started in 1992 with an increased growth rate as the main breeding objective. After four generations of selection for harvest weight, genetic gains of ~10% per generation were reported [2,3]. 

Genetic improvement programs for aquaculture species have been successfully established for increasing the productivity, for traits like growth and resistance against diseases [4,5]. However, one of the negative consequences of selective breeding is the accumulation of inbreeding, due to the use of related individuals for reproductive purposes [6]. As a consequence, a reduction in both the additive genetic variance and diversity is observed, as well as a decrease in the response to selection. Furthermore, inbreeding can result in inbreeding depression, defined as a reduction in fitness traits, including growth, survival and reproductive ability, due to the expression of detrimental recessive alleles given the existence of highly homozygous animals in the population [6,7]. Thus, monitoring and managing the inbreeding levels is critical in the operation of genetic improvement programs [8,9,10].

Pedigree-based inbreeding (F_ped_) is traditionally estimated by calculating the probability that an individual inherits two alleles that are identical-by-descendent (IBD). When F_ped_ is used, the founder animals in the pedigree are considered unrelated. This assumption fails to capture the actual relatedness among animals from the base population [11]. Thus, pedigrees errors and incomplete or missing information might lead to incorrect or biased inbreeding estimates [12]. Furthermore, F_ped_ is computed as an average expectation (i.e., probabilities) and does not consider the stochastic nature of recombination during meiosis and the finite number of chromosomes [13]. The development of genomic technologies, including dense single nucleotide polymorphism (SNP) panels, creates opportunities to estimate inbreeding from genomic-based approaches, e.g., by using a genomic relationship matrix to infer identity-by-state (IBS) [14] or through ROH [15].

ROH is defined as continuous DNA segments that are homozygous in a particular individual [16], as a potential consequence of not random mating, demographic history of consanguineous mating due to parents transmitting identical haplotypes to their offspring [17]. Therefore, ROH may provide a good measure of individual genome-wide autozygosity, which is the homozygote status generated by IBD alleles, that resulted from genetic drift, population bottleneck, the mating of close relatives and selection [13,18]. Furthermore, the identification and characterization of ROH can provide insights into population history, structure and demographics over time, allowing to distinguish between recent and ancient inbreeding [19,20]. Long ROH segments are indicative of recent IBD (>16 Mb = three generations), whereas short segments suggest ancient inbreeding (1 Mb = 50 generations). The sum of all these segments is suggested to be an accurate estimate of the actual inbreeding level of an individual [21]. However, it also possible that not all small size ROH are due to IBD, but to identity-by-status (IBS) due to low recombination rates in specific regions of the genome, high linkage disequilibrium in non-related ancestors and genetic drift.

Inbreeding studies using genome-wide data were previously reported in humans [16,22,23], livestock [24,25,26,27,28], swine [29,30,31], sheep [32], goats [33] and river buffalo [34]. Several studies have shown that ROH provides a better measure of individual autozygosity at the genomic level and the possibility to identify specific IBD regions [16,27,35]. A recent study reported ROH patterns in rainbow trout populations to show the impact on selection on the genetic diversity in farmed stocks [36]. However, studies aimed to characterize ROH patterns and comparisons between coefficients of inbreeding using different approaches are scarce in aquaculture species, due to the need of deep and complete pedigree information and dense genomic information, which most of the time is insufficient. The objectives of this study were to identify and characterize the ROH patterns, and to compare inbreeding coefficients estimated using genomic and pedigree information in three farmed Chilean coho salmon populations.

## 2. Methods 

### 2.1. Coho Salmon Populations and Genotypes

Two independent coho salmon populations, managed in two-year reproductive cycles (POP A and POP B) were used in this study and belong to the Pesquera Antares breeding program established in Chile in 1997. Both populations have undergone nine generations of selection for harvest body weight, since 1997 and 1998, POP A and B respectively. In addition, POP C is the progeny produced by mating sires from the seventh and dams from eighth generations of POP A and B, respectively. POP C was generated in 2013 to limit inbreeding levels, as suggested by Yáñez et al. [8]. The reproduction system, fish tagging and selection criteria of coho salmon populations were described previously [37,38].

Genomic DNA was extracted from fin clips of 88, 45 and 104 animals from POP A, B and C, respectively. The samples were genotyped using a 200 K Affymetrix Axiom^®^ myDesign Custom SNP Array developed by the EPIC4 coho salmon genome consortium (http://www.epic-4.org) and built by ThermoFisher Scientific (San Diego, CA, USA) [39]. A genotype quality control was performed in Plink v1.09 [40] using the following parameters to exclude markers: Hardy–Weinberg Equilibrium (HWE) *p*-value < 1 × 10^−6^, Minor Allele Frequency (MAF) <0.01 and call rate <0.90 for genotypes and samples. Furthermore, we retained only the SNP markers that commonly segregated among the three populations. Sampling protocols were approved by the Animal Bioethics Committee from Universidad de Chile (No. 08-2015) and all raw genotypic data are available at Figshare public repository (10.6084/m9.figshare.11931963).

### 2.2. Principal Components and Admixture Analysis

We used the software Plink v1.09 [40] to evaluate the genetic differentiation among the three coho salmon populations through principal component analysis (PCA). The first two PCAs were plotted using R scripts [41]. The population structure was also examined using a hierarchical Bayesian model implemented in STRUCTURE software v.2.3.4 [42]. We used three replicates of K values ranging from 1 to 12, running of 50,000 iterations and burn-in of 20,000 iterations. To choose the best K value we used the statistic ΔK [43]. 

### 2.3. Runs of Homozygosity 

Runs of homozygosity analysis was performed separately for all animals in each population using the R package detectRUNS [44]. The following constraints were applied to ROH detected: (i) the minimum number of SNPs included in an ROH was 50, (ii) the minimum length of an ROH was set at 1 Mb, (iii) the maximum distance between adjacent SNPs was 500 Kb, (iv) maximum missing genotypes allowed was 5, (v) density was at least 1 SNP per 50 kb and (vi) sliding windows approach was used to detect ROH for each genotyped animal at each marker position. ROH was classified into five-length classes: 1–2, 2–4, 4–8, 8–16 and >16 Mb, identified as ROH_1–2 Mb_, ROH_2–4 Mb_, ROH_4–8 Mb_, ROH_8–16 Mb_, and ROH_>16 Mb_, respectively. 

Each ROH size class represent the number of generations from common ancestry, estimated as: $E\left(L_{IBD-H}|gcA\right)=\frac{100}{2gcA},$ where $E\left(L_{IBD-H}|gcA\right)$ is the ROH segment length and *gcA* is the number of generations from the common ancestor [13]. Thus, we would expect that ROH_1–2 Mb_, ROH_2–4 Mb_, ROH_4–8 Mb_, ROH_8–16 Mb_, and ROH_>16 Mb_ are dating to approximately 50, 20, 12.5, 6 and 3 generations ago by considering that the 1 cM equals 1 Mb.

### 2.4. Inbreeding Coefficient

We estimated inbreeding coefficients using three different genomic methods and pedigree relationship matrix (F_PED_). Inbreeding coefficient based on runs of homozygosity (F_ROH_) was estimated for each animal based on all ROHs (ROH_ALL_) and the ROH distribution of five different lengths (ROH_1–2 Mb_, ROH_2–4 Mb_, ROH_4–8 Mb_, ROH_8–16 Mb_, and ROH_>16 Mb_), as follows [16]:

(1) $$F_{\mathrm{ROH}}=\frac{L_{ROH}}{L_{AUTO}},$$

where L_ROH_ is the sum of ROH lengths and L_AUTO_ is the total length of the genome covered by the genome-wide SNP panel used, assumed to be 1685.79 Mb. 

The F_HOM_ was calculated based on genome-wide homozygous excess due to inbreeding as follows [45]:

(2) $$F_{\mathrm{HOM}}=\frac{O_{hom}-E_{hom}}{N-E_{hom}},$$

where $O_{hom}$ is the observed number of homozygous markers in each individual, $E_{hom}$ is the expected number of homozygous markers under the Hardy-Weinberg equilibrium calculated from the allele frequencies estimated based on the sample and N is the total number of markers. 

The F_GRM_ was estimated using the genomic relationship matrix (GRM) [14], as follows:

(3) $$G=\frac{ZZ^{\prime }}{2\sum_{i=1}^{n}p_{i}(1-p_{i})},$$

where Z is a genotype matrix that contains the 0–2p values for homozygotes, 1–2p for heterozygotes, and 2–2p for opposite homozygotes, p is the allele frequency of SNP *i*. The diagonal elements of the matrix G represent the relationship of the animal *j* with itself, thus, the genomic inbreeding coefficient is calculated as 2G*_jj_*–1. 

Pedigree-based inbreeding coefficients were estimated using the software INBUPGF90 [46]. The pedigree information used was provided by Pesquera Antares breeding program in Chile, for all animals born between 1998 and 2014, 1997 and 2013 and 1998 and 2013 for POP A, B and C respectively. Pearson correlation between genomic- and pedigree-based inbreeding coefficients were estimated within and across all populations using function cor.test in R [41]. 

## 3. Results

### 3.1. Quality Control and Genomic Population Structure

From an initial number of 135,500 markers, a total of 102,129 passed in the QC and were shared among the three populations. A number of ~30 K, 21 K and 18 K markers for POP A, B and C, respectively were removed, most of them due to MAF criteria. In addition, a number of markers ranging from 3.2 K to 14.9 K were removed to select only common markers segregating across all three populations (Supplementary File 1).

**Figure 1:**
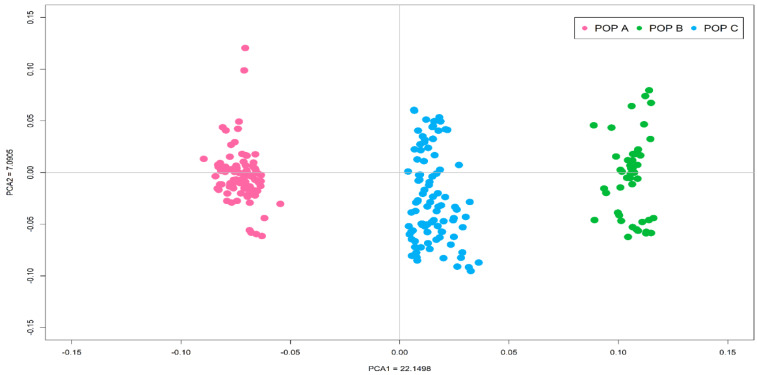
Principal component analysis of the autosomal genotypic data of three coho salmon populations.

In the PCA analysis, the first two eigenvectors, together, accounted for 29.2% of the total genetic variation and revealed three stratified populations (Figure 1). PCA1 included 22.15% of the total genetic variation and generated the principal clusters to differentiate the three coho salmon populations, whereas PCA2 explained the variation present within each population. 

The best K-value for admixture analysis was selected after performing several runs of MCMC for each K-value (ranging from 1 to 12), based on the statistic ΔK the best K-value was suggested to be *K* = 4. These results indicated that for few individuals of POP A and POP B the proportion of ancestry come from a single cluster (green and yellow, respectively), and most of animals shared a proportion of their genome with each other, probably due to the similar origin of the source populations. In addition, Supplementary File 2 indicate a higher admixture level for most of animals from POP C, due to the recent cross between POP A and B to generate this population. 

### 3.2. Distribution of Runs of Homozygosity

We identified ROH in all animals for coho salmon POP A and B, and in 103 out of 104 individuals for POP C. A total of 3250, 1497, and 266 ROH and an average number of 36.93 ± 7.13, 35.65 ± 8.64, 2.65 ± 1.27 ROH per animal were identified for POP A, B and C, respectively. The mean ROH length was 6.47 ± 7.39, 7.17 ± 7.69 and 2.58 ± 2.07 Mb for POP A, B and C, respectively (Table 1) and the longest segment identified was 61.82 Mb, found in chromosome 2 for POP B (Supplementary File 3). The ROH analysis for different length classes suggests that for the three coho salmon populations the genome is mostly composed of a high number of short segments (ROH_1–2 Mb_, ROH_2–4 Mb_). No segment was found for ROH_>16Mb_ in POP C.

**Figure 2:**
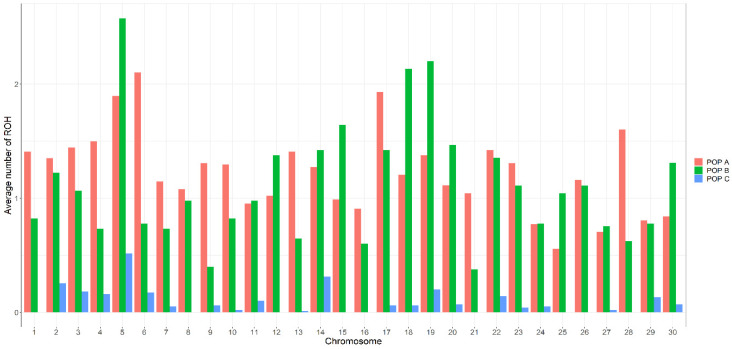
Distribution of the average number of ROH per individual for each chromosome in three coho salmon populations.

**Figure 3:**
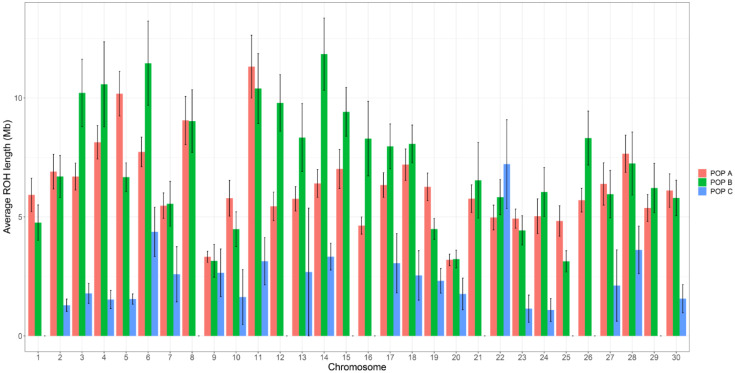
Average ROH length and standard error bars for each chromosome in three coho salmon populations.

The average number of ROH identified differs between chromosomes and in low magnitude between populations. For POP A and POP B, most of the chromosomes resulted in the average number of ROH between one and two ROH per animal and more than two ROHs in chromosome Okis6 for POP A and Okis5, Okis18 and Okis19 for POP B, whereas for POP C all chromosomes have less than one ROH per individual (Figure 2). The average ROH length also differs between chromosomes and the population. POP A has two chromosomes (Okis5 and Okis11) with ROH segments greater than 10 Mb. POP B has five chromosomes (Okis3, Okis4, Okis6, Okis11 and Okis14) with ROH segments greater than 10 Mb; while all chromosomes in POP C have ROH segments smaller than 7 Mb (Figure 3).

**Figure 4:**
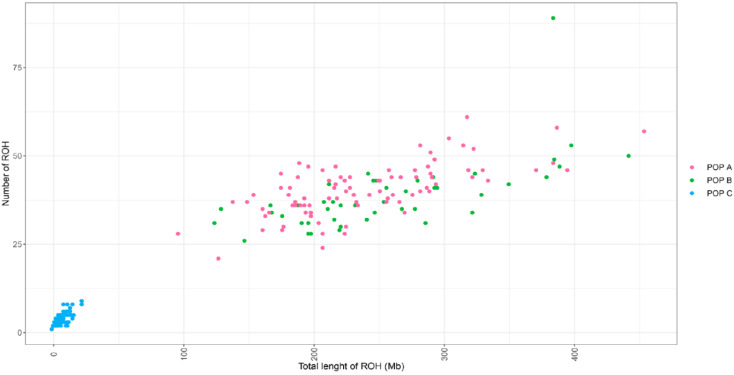
Relationship between the number of runs of homozygosity (ROH) and total length of ROH (Mb) per individual from each population.

Figure 4 shows the relationship between the total number of ROH and the total length of ROH for each animal across the three populations. A considerable difference between POP C and POP A or B was found. For POP C, all animals have a small number of ROH (<8) with total length <25 Mb, whereas most individuals in POP A and B have at least 20 ROHs with a total length >100 Mb, with some individuals with segments covering more than 300 Mb. The number of ROHs and segment length per animal and per chromosome are shown in Supplementary File 3. The high number of segments >10 Mb in Okis5, Okis6 and Okis28, especially for POP A and B, suggests recent events of inbreeding, whereas the small segments as in Okis20 for POP A and B, and for most of the chromosomes for POP C, suggests more ancient inbreeding. 

### 3.3. Genomic- and Pedigree-Based Inbreeding

**Figure 5:**
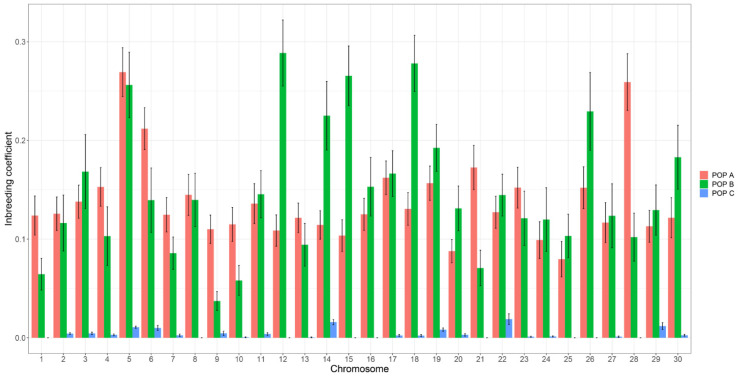
Distribution of average inbreeding coefficients estimated using ROH for each chromosome in three coho salmon populations. Standard error bars were computed among individuals from the same population.

We used four different methods to estimate the inbreeding coefficient, from the information of 102 K markers and pedigree data (Table 2). The average inbreeding coefficient estimated using ROH was different between ROH classes, the values decreased when the ROH length segments increased for all populations. The mean value for FROH_ALL_ was the same for both POP A and B (0.142 and 0.152, respectively), but it was significantly different (*p* < 0.05) for POP C (0.004) when compared to POP A or B. The F_HOM_ resulted in the lowest inbreeding values ranging from −0.036 to −0.105 for POP A and C, respectively. The mean value for F_GRM_ was different (*p* < 0.05) between the three populations, the highest and lowest values were reported for POP B and C, respectively, whereas the F_PED_ value was not different between POP A and B, but was significantly lower for POP C (0.002, *p* < 0.05). Additionally, we estimated the inbreeding coefficient based on the ROH per chromosome (Figure 5). POP A and B had the most chromosomes with inbreeding values higher than 0.2, as in Okis5, Okis6 and Okis28 for POP A, and Okis5, Okis12, Okis14, Okis18 and Okis26 for POP B, whereas lower values were found for POP C and for most of the chromosomes the inbreeding was equal to zero. 

The Pearson correlation between different genomic methods to estimate the inbreeding coefficient suggested a high positive correlation (>0.82, *p* < 0.001) for POP A and POP B (Supplementary File 4 and 5, respectively). Correlation between different ROH length classes decreased in function with the comparison between shorter and longer segments, e.g., highest correlation between ROH_1–2 Mb_ and ROH_2–4 Mb_ and lowest between ROH_1–2 Mb_ and ROH_>16Mb._ The lowest correlation values among genomic methods was reported between ROH_>16Mb_ and both ROH_HOM_ and ROH_GRM_. In addition, for POP A and POP B low correlation values were found, respectively, ranging from 0.35 to 0.39 (*p* < 0.01), between genomic methods and F_PED_. 

Different patterns of correlations were observed for POP C, compared to POP A and B, probably due to the low inbreeding level of this recently admixed population. Medium to high positive correlation was reported between the ROH classes (0.54 to 0.94, *p* < 0.001), and a correlation equal to unity was observed between ROH_HOM_ and ROH_GRM._ For other correlations, small values (ranged from 0.28 to 0.34) or not different from zero were observed (Supplementary File 6).

The across-populations Pearson correlation coefficients between different genomic- and pedigree-based inbreeding estimates ranged between 0.56 and 1.00, with the minimum and maximum correlation values found between F_HOM_—F_PED_ and FROH_1–2 MB_—FROH_2–4 MB_—FROH_4–8 MB_ comparisons, respectively. All correlation values among inbreeding coefficient calculated for all individuals across populations are shown in Supplementary File 7. 

## 4. Discussion

The first two principal components explained more than 29% of the total genetic variation for the three populations studied, which were separated into three different clusters. The admixture results are in agreement with the recent event of hybridization of POP A and B to generate POP C, where the genetic differentiation between POP A and B may have been be partly generated by differences in the base population, which can have a pronounced effect on allele frequencies [47]. In addition, considering that POP A and B have been independently selected by at least eight generations each, differences in the selection processes, as well as the environmental conditions and drift, may have influenced the differences observed in Supplementary File 2. 

The ROH patterns seem to be differentially distributed within specific genomic regions, same as the inbreeding values between chromosomes. The highest autozygosity, e.g., in chromosome Okis5 and Okis6 for POP A and B, is likely the consequence of artificial selection [28], considering that these populations have been under genetic selection for harvest weight for at least eight generations. A ROH study in humans [48] suggested that the homozygosity segments are more common in regions with high linkage disequilibrium (LD) and low recombination rates. Thus the highest mean levels of LD found in Okis5 and Okis6 in animals from the same populations [39] are in accordance with the two chromosomes with the larger ROH sizein the present study. 

Differences in the number of ROH and segment length was observed within and across populations. The greater number of ROH in POP A compared to POP B can be due to higher sample size in the former, considering that the average number of ROH per animal had small variation between populations. The differences in average ROH length may be due to recent inbred mating or other demographic processes along the time in the different populations, which can generate different distribution of long and short ROH segments [49]. Furthermore, the intense artificial selection might have altered the allele frequency and increase the IBD haplotypes and created long ROH in specific regions of the genome [28]. In contrast, the shorter segments and smaller number of ROH in POP C when compared against both POP A and B may be the result of recent population admixture between these populations. Furthermore, animals from the same population might have the same total ROH lengths but a variable number of segments, which is probably the result of different distances from common ancestors [25]. Interestingly, for both POP A and B, the length class ROH_2–4 Mb_ has more ROH than ROH_1–2 Mb._ These differences can be due to the criteria adopted to identify ROH or an inherent characteristic of these populations. There is no consensus on the best parameters to characterize ROH patterns [36]; thus, here we used the minimum number of 50 SNPs and the length of 1 Mb to define a ROH segment. We chose the current parameters due to the historical demographics of coho salmon in Chile. The ROH_2–4 Mb_ should date from about 20 generations ago (approximately 40 years considering the generation interval of two years), which corresponds to the introduction of coho salmon in Chile at the end of the 1970s, to begin the establishment of Chilean brood stocks [2,39].

Based on information of ROH length it is possible to infer the number of generations for inbreeding events [50]. The ROH due to ancient origin tend to be shorter, e.g., ROH_1–2 Mb_, ROH_2–4 Mb_ and ROH_4–8 Mb_ date from 50, 20 and 12.5 generations ago, respectively. In contrast, recent ROH are longer, due to the small probability of breaking down the segments that are IBD by means of recombination events. Thus, the ROH_8–16 Mb_ and ROH_>16 Mb_ are dated to six and three generations ago, respectively [22,50]. For both POP A and B it was possible to identify short and long segments in most of the animals analyzed, whereas in the POP C a small number of animals (*n* = 7) presented ROH_8–16 Mb_ and none ROH_>16 Mb_. 

In recent years, some studies have investigated different genomic methods to estimate inbreeding coefficients in livestock [11,25,28,35,51,52], pigs [29,30,53,54] and goats [55,56,57]. Recently, ROH studies in rainbow trout [36] and turbot [58] reported different average ROH length for these aquaculture species; 4 Mb and 0.77 Mb rainbow trout and turbot, respectively. These differences can be explained due to the genome size, that is 3.5-fold smaller in turbot [58], and also based in different demographic history in the populations assessed. The greater average ROH segments found in the present study (6.6 Mb and 7.2 Mb for POP A and POP B, respectively) are most likely due to high selection intensity during almost eight generations in these coho salmon population and the mate allocation strategies that resulted in high level of inbreeding, and consequently longer ROH segments. However, this is the first study aimed at characterizing the ROH patterns and comparing different genomic- and pedigree-based methods to estimate inbreeding coefficients in farmed coho salmon populations. The pedigree-based inbreeding coefficient, is a simple method that requires recording genealogy information, but does not account for the autozygosity differences among animals with the same inbreeding history. Furthermore, considering that the founder animals are unrelated pedigree-based inbreeding estimates may lead to underestimation of autozygosity levels [11]. In contrast, with the availability of high-density SNP information, inbreeding can be accurately measured, even without pedigree information [15]. ROH can provide a better estimation of whole-genome autozygosity levels, by identifying IBD segments with great accuracy [51], and based on length of the ROH segments the ancient and contemporary inbreeding can be reported [29]. 

A comparison of inbreeding coefficients, showed that F_GRM_ gave the highest values, especially for B and C, probably because the alleles IBD and IBS are not differentiated for F_GRM_ [11]. This result is in agreement with results previously found in humans, cattle, and simulation studies [11,15,16]. F_HOM_ resulted in negative inbreeding values for all populations, suggesting that the individuals have lower levels of homozygosity than expected in the reference population under Hardy-Weinberg equilibrium [59] and underestimated values should be expected [60]. The F_PED_ for POP A and POP B were smaller than values estimated using FROH_ALL_ and F_GRM,_ but are in accordance with the values estimated for the same populations using previous generations [8,10]. The F_PED_ can be easily underestimated when pedigree information of less than 20 generations is used [60]. The difference between F_ROH_ and F_PED_ could also be due to the unknown pedigree information before base population, which in practical terms means that inbreeding levels for founding animals were not zero.

ROH can be identified for each genotyped animal, allowing to detect specific location in the genome with high levels of autozygosity, i.e., along chromosomes [61]. In this regard, we used the Pearson correlation to evaluate the agreement between F_ROH_ and other approaches to estimate genomic inbreeding (F_GRM_ and F_HOM_)_._ Correlations between F_GRM_ and F_ROH_ decreased while decreasing size of ROH classes probably due to that G matrix that is based in individual loci, whereas F_ROH_ is based on chromosomal segments, and the F_GRM_ cannot distinguish between alleles that are IBD and IBS [62]. Another reason which may explain this pattern is that ROH is the sum of large and short segments, and ROH classes of smaller size could be more informative on the homozygosity status across the genome when compared to classes of larger ROH segments. It has also been previously suggested that both F_GRM_ and F_HOM_ are strongly dependent on allele frequencies, especially for populations with divergent allele frequencies (high level of heterozygosity and some rare alleles with small frequency), which can result in misleading IBD [51,63]. The high correlations (>0.80) found between F_ROH_ and other genomic inbreeding estimates for POP A and B, and moderate to strong positive correlation between genomics methods used to estimate inbreeding coefficients has been reported for different species [27,29,62,64], suggesting that the extent of homozygosity in a genome can be accurately used to predict the proportion of the genome that is IBD. In contrast, we found low correlation between F_ROH_ in different length classes and F_GRM_ and F_HOM_ for the POP C, which makes sense considering that this is a recently admixed population, and the correlation might be affected by the average degree of actual homozygosity of population [27,35].

The genomic-based inbreeding method correlated moderately or poorly with pedigree data, showing values lower than 0.39. Similarly weak or no correlation was reported for cattle [24,51,52], whereas a moderate to strong positive correlation was described by some authors [15,16,35,65]. An increase in the correlation between genomic- and pedigree-based inbreeding as the pedigree depth increases is expected [24]. Here we used the complete pedigree information of nine generations for both POP A and POP B, whereas for POC C, a pedigree depth of eight generations was used. In a previous pedigree-based inbreeding study using the same broodstock population of POP A (7th generation) and POP B (8 th generation), an increasing tendency for inbreeding values in the last four generations was reported for both populations [8] and a continued inbreeding accumulation until 9 th generation used in our study is well-known. Thus, we expected a higher correlation between long ROH segments (ROH_8–16 Mb_, and ROH_>16 Mb_) and F_PED_ values. The weak or no correlation may be explained by the depth of pedigree records [61], incorrect or incomplete pedigree information [51]. The F_PED_ assumed that the founder individuals are unrelated [11], does not consider the stochastic nature of recombination and the persistence of ancestral short segments through time, due to the lack of recombination in specific regions [27]. These facts suggest that the F_PED_ may not reflect true inbreeding values. In addition, the correlation between genomic and pedigree-based inbreeding can be also affected by the parameters to determine ROH [35,36,66] and the population sample size [15,24]. Here we chose the parameters to identify ROH segments based on those reported in previous studies [27,36,52,65,67,68]. Regarding sample size, the POP B is the population with the smallest sample size (*n* = 45); however, was the only one that resulted in significant correlations between genomic- and pedigree-based inbreeding levels. Moreover, various studies on the characterization of ROH patterns in production species used similar or smaller sample size [26,35,36,69].

A relatively large effective population size (Ne), ranged from 50 to 200, is recommended to maintain the control of inbreeding in the medium-term [70]. However, decline in the historical Ne was reported for animals from the same population as POP A [39]. The reduction may be due to the prioritization of genetic gain using high selection pressure without putting strong control on the family contribution for each generation [8]. Consequently, mating close relatives is more probable, which results in a high level of inbreeding and the creation of long ROH segments for both POP A and B. Therefore, to increase the effective population size and to limit the inbreeding level [8], POP C was generated. According to our results, this strategy was effective in reducing the inbreeding levels and changing the patterns of ROH, clearly differentiating from POP A and B. These results are in accordance with some studies [15,35,55,57] that suggest that high heterogeneity populations due admixture or crossbreeding lines contributed to the breakdown of long homozygous segments and reduced the inbreeding levels in captive populations. 

## 5. Conclusions

We found different numbers and lengths of runs of homozygosity in three coho salmon populations included in the study. Moreover, the inbreeding coefficient estimated using genomic or pedigree-based methods varied among populations. The higher correlations between genomic-based inbreeding methods, except for POP C and most likely due to recent admixture history, suggests that genomic approaches are more accurate to estimate autozygosity levels, and thus, must be used as the methods of choice when pedigree information is inaccurate, incomplete or unavailable.

## Figures and Tables

**Table 1:** Total number of runs of homozygosity (ROH) (N) per class, the average number of ROH per individual (N Mean) and average length (Mb) considered all ROH and by classes for three coho salmon populations.

Class	POP A				POP B				POP C		
	N	N Mean	Length		N	N Mean	Length		N	N Mean	Length
**ROH_ALL_**	3250	7.39 (3.75)	6.47 (7.39)		1497	6.65 (3.84)	7.17 (7.69)		266	0.54 (0.85)	2.58 (2.07)
**ROH_1–2 Mb_**	847	9.63 (3.16)	1.46 (0.29)		341	7.58 (3.60)	1.46 (0.29)		158	1.60 (1.06)	1.40 (0.26)
**ROH_2–4 Mb_**	937	10.65 (3.28)	2.89 (0.58)		400	8.89 (4.33)	2.84 (0.53)		58	0.59 (0.69)	2.86 (0.58)
**ROH_4–8 Mb_**	680	7.73 (2.73)	5.59 (1.07)		310	6.89 (3.74)	5.56 (1.03)		43	0.43 (0.59)	5.11 (1.14)
**ROH_8–16 Mb_**	463	5.26 (2.26)	11.31 (2.32)		260	5.78 (3.06)	11.45 (2.25)		7	0.07 (0.26)	11.54 (0.04)
**ROH_>16 Mb_**	323	3.67 (1.75)	24.93 (7.68)		186	4.13 (2.58)	23.69 (8.08)		0	0	-

Standard deviation in brackets.

**Table 2:** The number of individuals (N), mean and standard deviation (SD) of inbreeding coefficients using runs of homozygosity (FROH) for different ROH length, based on the excess of homozygosity (F_HOM_), genomic relationship matrix (F_GRM_) and pedigree-based relationship matrix (F_PED_) for each coho salmon population.

Class	POP A				POP B				POP C		
	N	Mean	SD		N	Mean	SD		N	Mean	SD
**FROH_1–2 Mb_**	88	0.142 ^a^	0.038		45	0.152 ^a^	0.044		103	0.004 ^b^	0.003
**FROH_2–4 Mb_**	88	0.133 ^a^	0.038		45	0.145 ^a^	0.044		73	0.003 ^b^	0.003
**FROH_4–8 Mb_**	88	0.115 ^a^	0.037		45	0.129 ^a^	0.043		47	0.002 ^b^	0.002
**FROH_8–16 Mb_**	88	0.090 ^a^	0.036		45	0.104 ^a^	0.043		7	0.000 ^b^	0.002
**FROH_>16 Mb_**	85	0.054 ^a^	0.028		45	0.062 ^a^	0.036		-	-	-
**FROH_ALL_**	88	0.142 ^a^	0.038		45	0.152 ^a^	0.044		103	0.004 ^b^	0.003
**F_HOM_**	88	−0.036 ^b^	0.048		45	−0.069 ^a^	0.056		104	−0.105 ^c^	0.011
**F_GRM_**	88	0.145 ^b^	0.037		45	0.193 ^a^	0.040		104	0.051 ^c^	0.009
**F_PED_**	88	0.071 ^a^	0.021		45	0.076 ^a^	0.027		104	0.002 ^b^	0.012

Different letters in each row indicate statistical significance for the comparison of methods for inbreeding estimation within populations (*p* < 0.05).

## Supplementary Materials

The following are available online at https://www.mdpi.com/2073-4425/11/5/490/s1, Table S1: Summary of results from quality control of SNP genotypes. Figure S1: Admixture clustering of the three coho salmon population for *K* = 4. Figure S2: Runs of homozygosity patterns for all chromosome coho salmon populations. Figure S3: Scatterplots and Pearson correlation of inbreeding coefficients for POP A. Figure S4: Scatterplots and Pearson correlation of inbreeding coefficients for POP B. Figure S5: Scatterplots and Pearson correlation of inbreeding coefficients for POP C. Figure S6: Scatterplots and Pearson correlation of inbreeding coefficients across coho salmon populations.
